# Physical health-related quality of life at higher achieved hemoglobin levels among chronic kidney disease patients: a systematic review and meta-analysis

**DOI:** 10.1186/s12882-020-01912-8

**Published:** 2020-07-08

**Authors:** Murilo Guedes, Camila R. Guetter, Lucas H. O. Erbano, Andre G. Palone, Jarcy Zee, Bruce M. Robinson, Ronald Pisoni, Thyago Proença de Moraes, Roberto Pecoits-Filho, Cristina P. Baena

**Affiliations:** 1 Pontifícia Universidade Católica do Paraná, Imaculada Conceição, 1155, Curitiba, PR 80215-901 Brazil; 2 Universidade Federal do Paraná, Curitiba, Paraná Brazil; 3 Arbor Research Collaborative for Health, Ann Arbor, MI USA

**Keywords:** Anemia, Chronic kidney disease, Health-related quality of life

## Abstract

**Background:**

The impact of anemia treatment with erythropoietin stimulating agents (ESA) on health-related quality of life (HRQOL) in chronic kidney disease (CKD) patients is controversial, particularly regarding optimal hemoglobin (Hb) target ranges.

**Methods:**

We conducted a systematic review and meta-analysis of observational studies and randomized controlled trials (RCT) with ESA to estimate the effect of different achieved Hb values on physical HRQOL and functionality. We searched PubMed, EMBASE, CENTRAL, PEDro, PsycINFO and Web of Science databases, until May 2020. Two authors independently extracted data from studies. We included observational and RCTs that enrolled CKD patients undergoing anemia treatment with ESA with different achieved Hb levels among groups. We excluded studies with achieved Hb < 9 g/dL. For the meta-analysis, we included RCTs with control groups achieving Hb 10–11.5 g/dL and active groups with Hb > 11.5 g/dL. We analyzed the standardized mean difference (SMD) between groups for physical HRQOL.

**Results:**

Among 8496 studies, fifteen RCTs and five observational studies were included for the systematic review. We performed the meta-analysis in a subset of eleven eligible RCTs. For physical role and physical function, SMDs were 0.0875 [95% CI: − 0.0025 – 0.178] and 0.08 [95% CI: − 0.03 – 0.19], respectively. For fatigue, SMD was 0.16 [95% CI: 0.09–0.24]. Subgroup analysis showed that trials with greater achieved Hb had greater pooled effects sizes — 0.21 [95% CI: 0.07–0.36] for Hb > 13 g/dL vs. 0.09 [95% CI: 0.02–0.16] for Hb 11.5–13 g/dL. Proportion of older and long-term diabetic patients across studies were associated with lower effect sizes.

**Conclusion:**

Achieved hemoglobin higher than currently recommended targets may be associated with small but potentially clinically significant improvement in fatigue, but not in physical role or physical function. Younger and non-diabetic patients may experience more pronounced benefits of higher Hb levels after treatment with ESAs.

## Background

A meaningful shift to more patient-centered care has been emphasized in the management of chronic kidney disease (CKD) [1]. This has been recently highlighted in different initiatives aiming to improve patient-reported outcomes (PRO) in Nephrology, such as the Standardized Outcomes Nephrology (SONG) and The Kidney Health Initiative (KHI) [2, 3].CKD has been associated with an important burden on health-related quality of life (HRQOL), and outcomes such as limitations to perform daily activities, poor physical functioning, and fatigue have been highly valued and prioritized by patients [4–6].

Anemia is found in up to 40% of patients with advanced ND-CKD and has been associated to higher mortality and morbidity [7, 8]. In fact, lower hemoglobin (Hb) has been shown to be one of the potential modifiable causal factors for CKD associated fatigue, which is a multi-dimensional concept with diverse causes, ranging from social, psychological and clinical factors, that is perceived as one of the most important outcomes for CKD patients and caregivers [9, 10]. Current recommendations for clinical management of anemia show some variation across different society guidelines. KDIGO recommendations consist in targeting Hb levels of 10–11.5 g/dL in patients undergoing erythropoietin stimulating agents (ESA) treatment [11]. According to the European Renal Best Practice (ERBP), treatment should target 10–11 g/dL ranges [12].

Such recommendations are based on results of major randomized controlled trials (RCT) of ESAs, which have not only failed to show benefits in mortality in CKD population, but could also result in increased risks of cardiovascular events when used to achieve Hb normalization [13]. In addition, the potential benefits for HRQOL are controversial particularly for targets of Hb above 9 g/dL [14–16]. Previous systematic reviews suggested that the effect of anemia correction on HRQOL may be small, although the estimates were highly heterogeneous [17–22]. Particularly, one systematic review restricted to dialysis patients suggested that the potential benefits on fatigue of anemia correction beyond 10–12 g/dL levels are limited [23], while a more recent meta-analysis comparing ESA targets concluded that HRQOL changes following ESA treatment are not clinically meaningful [21]. These systematic reviews, however, do not distinguish between achieved vs. aimed targets and therefore have limitations to the efficacy of higher achieved Hb levels on HRQOL. Based on the heterogeneity of the finding in this area, KDIGO guidelines recommend the individualization of Hb targets aiming better HRQOL outcomes, suggesting subgroups for higher benefit, such as younger patients, despite the lack of evidence [11].

Previous studies have suggested that younger patients with fewer comorbidities may benefit more from higher Hb levels in terms of HRQOL, given that the threshold for manifestations of limitations may depend on basal level of activities and the degree of exposure to chronic debilitating conditions [17]. Based on these findings, individuals that are more functional may experience more pronounced benefits of higher Hb levels after treatment with ESAs. In this sense, along the continuum of physical dysfunction [24–27], the comorbidity-related loss of functionality may represent a turning point when the potential effects on physical HRQOL of achieving higher Hb levels may not translate into further gains. Consequently, in this model, Hb targets should be dependent on the population in which they are pursued, rather than being absolute across distinct patients [11].

We therefore designed a systematic review and meta-analysis on the efficacy of Hb achievement and its impact on physical HRQOL. Specifically, we hypothesized that: i) achieved Hb levels greater than recommended by current clinical practice (for treatment with ESAs) are associated with better physical HRQOL; ii) patients with better functionality are more likely to benefit from higher Hb ranges after treatment with ESAs; iii) variables associated with worse functionality among populations explain part of the heterogeneity among studies. Therefore, we conducted a systematic review and meta-analysis to evaluate the impact of different achieved Hb levels with ESA treatment on physical HRQOL and functionality among CKD patients.

## Methods

### Selection criteria

We conducted a systematic review and meta-analysis according to PRISMA guidelines and MOOSE recommendations for meta-analysis of observational studies [28, 29] . We reviewed studies among CKD patients with anemia reporting outcomes in physical HRQOL or functionality published in English, Spanish or Portuguese. Physical HRQOL was assessed by scales for which there was at least one study referenced either in the original study report or in the study protocol reporting on reliability or validity. For the meta-analysis, studies were eligible if they reported on scales consistent with the Short-Form (SF-36), including SF-36 subscales, KDQOL-36 and KDQ. Functionality was evaluated either by activities of daily living (ADL) instruments or Karnofsky status.

For experimental studies, inclusion criteria consisted of adult populations treated for anemia with ESA exposed to different Hb targets. Exclusion criteria included placebo-controlled groups without ESA rescue strategies reported, i.e., placebo groups without a rescue ESA dose for those with achieved Hb ≤ 9 g/dL, studies with control group achieving hemoglobin ≤9 g/dL, hospitalized patients, pediatric or transplant populations, quasi-experimental studies, pre- and post-intervention studies and iron treatment RCTs.

Eligibility criteria for observational studies included adult CKD populations, hemoglobin levels or ESAs as main exposure, cohort studies and outcomes in physical domains of HRQOL or functionality. For physical HRQOL outcomes, we included only studies reporting estimates adjusted for a minimum set of confounders, including any group of comorbidities, age and eGFR. For functionality outcomes we did not exclude reports with unadjusted estimates. Studies in pediatric, transplant or hospitalized populations, cost-effectiveness, reviews, letters, case-reports, case-series and studies with less than 100 patients were excluded.

### Search strategy

The search for eligible studies was performed using the databases MEDLINE, EMBASE, CENTRAL, PEDro, PsycINFO and Web of Science, until May 2020. A combination of indexing terms and free text words in each database was used to build search strategies. The detailed search strategies are presented in the Supplementary Material. Grey literature was searched by review of conference abstracts.

### Data extraction

Two authors independently reviewed titles, abstracts and full reports to determine study inclusion. Disagreements in any phase were solved through discussion and a third author was consulted when needed. Two authors independently extracted data from eligible studies according to a pre-defined structure for data collection.

When more than one physical HRQOL assessment was performed during follow-up, we extracted the last reported value. Different reports from the same RCTs were included if additional information on physical HRQOL was provided. For studies with three arms - two arms comparing different targets with ESAs and one arm with placebo without rescue strategy - we included information only for the ESA arms.

### Risk of Bias

Risk of bias was assessed independently by two authors. For experimental studies, the Cochrane Tool for Bias Assessment was applied. For observational studies, the risk of bias was evaluated by the modified version of NewCastle Ottawa scale (NOS) for cohort studies [30, 31].

### Clinical assumptions

For RCTs, we assumed that achieved Hb between 10 g/dL and 11.5 g/dL in the subgroups of patients randomized to lower targets would represent current recommended clinical practices, based on KDIGO guidelines [11]. Consequently, achieved Hb greater than 11.5 g/dL in active groups are assumed to represent strategies higher than current recommendations. Moreover, we excluded studies with control group reporting mean achieved Hb lower than 10 g/dL, assuming that for these patients the clinical benefits of anemia treatment are established [11]. Therefore, to be eligible, studies had to report: 1) achieved hemoglobin values in lower target arms within the range of 10–11.5 g/dL, reflecting current recommended targets and 2) achieved hemoglobin values on higher target arms greater than 11.5 mg/dL, thereby avoiding group overlap. Finally, we defined the proportion of patients with diabetic nephropathy reported by each trial as representative of long-term complicated diabetes, which we considered a proxy for higher risk of lower functionality. We included studies in the meta-analysis according to their achieved hemoglobin values at the time of physical HRQOL assessment in the study. When studies evaluated physical HRQOL with more than one instrument, we chose to include data on KDQOL/SF-36 whenever possible.

### Statistical analysis

We performed a meta-analysis of standardized mean difference (SMD) between intervention groups, using Hedges’ g, from RCTs that reported sufficiently detailed data on change from baseline physical HRQOL [32] . A minimal important difference (MID) in scales was defined as 3 points on SF-36 and 0.5 for KDQ [33, 34]. For SMD, a small difference was defined as 0.2–0.5 points [35]. The pooled effect was estimated as a weighted standardized mean difference (SMD) for the change from baseline in scores of physical domains HRQOL between higher achieved hemoglobin vs. lower achieved hemoglobin groups in each study.

Fatigue reports from SF-36 and KQD vitality scales were analyzed together, as they have been shown to measure same fatigue dimensions [36]. We imputed standard deviations from incomplete reports, according to previously reported methods [37–39]. Sensitivity analyses were carried out. For this, we excluded the subset of studies that required imputation and rerun the analyses, checking for differences in the pooled estimates. Furthermore, we reanalyzed only the subset of studies reporting outcomes on SF-36. We undertook separate analysis for subdomains of SF-36 physical dimension - physical role, physical function and vitality. For presenting the results and for comparisons with MID boundaries defined for SF-36, we back-transformed the SMD to mean differences in SF-36 scale by multiplying the effect size in SMD by the median standard deviation of SF-36 for the outcome subdomain from studies presenting results with this instrument [40].

Random effects models were used to estimate the pooled effect. Between study variance was estimated by the DerSimonian-Laird estimator and the Hartung–Knapp adjustment was used [41–43]. Heterogeneity between studies was measured by I-square and Cochran’s Q test was run to test the null hypothesis of homogeneity considering an alpha level of 0.10. We explored heterogeneity by mixed effects meta-regression and subgroup analyses for outcomes with more than 10 studies contributing to pooled effect estimate [44]. The a priori specified variables to be tested in meta-regression were age, diabetic nephropathy proportion, and follow-up period in weeks. For subgroup analysis, achieved hemoglobin in active groups within trials (categorized as Hb equal to 13 g/dL), renal replacement therapy (RRT) and blinding for both intervention and HRQOL assessments were explored. Treatment effects-subgroups interaction was evaluated by Cochran Q test for heterogeneity [45]. Publication bias was evaluated using funnel plots and small study effects were assessed by the Egger test, for outcomes with more than 10 studies contributing to analysis [46, 47]. Trim and fill method was performed to adjust for small study effects [48]. All analyses were conducted using R software version 3.5.1 with the package “meta”.

## Results

**Fig. 1:**
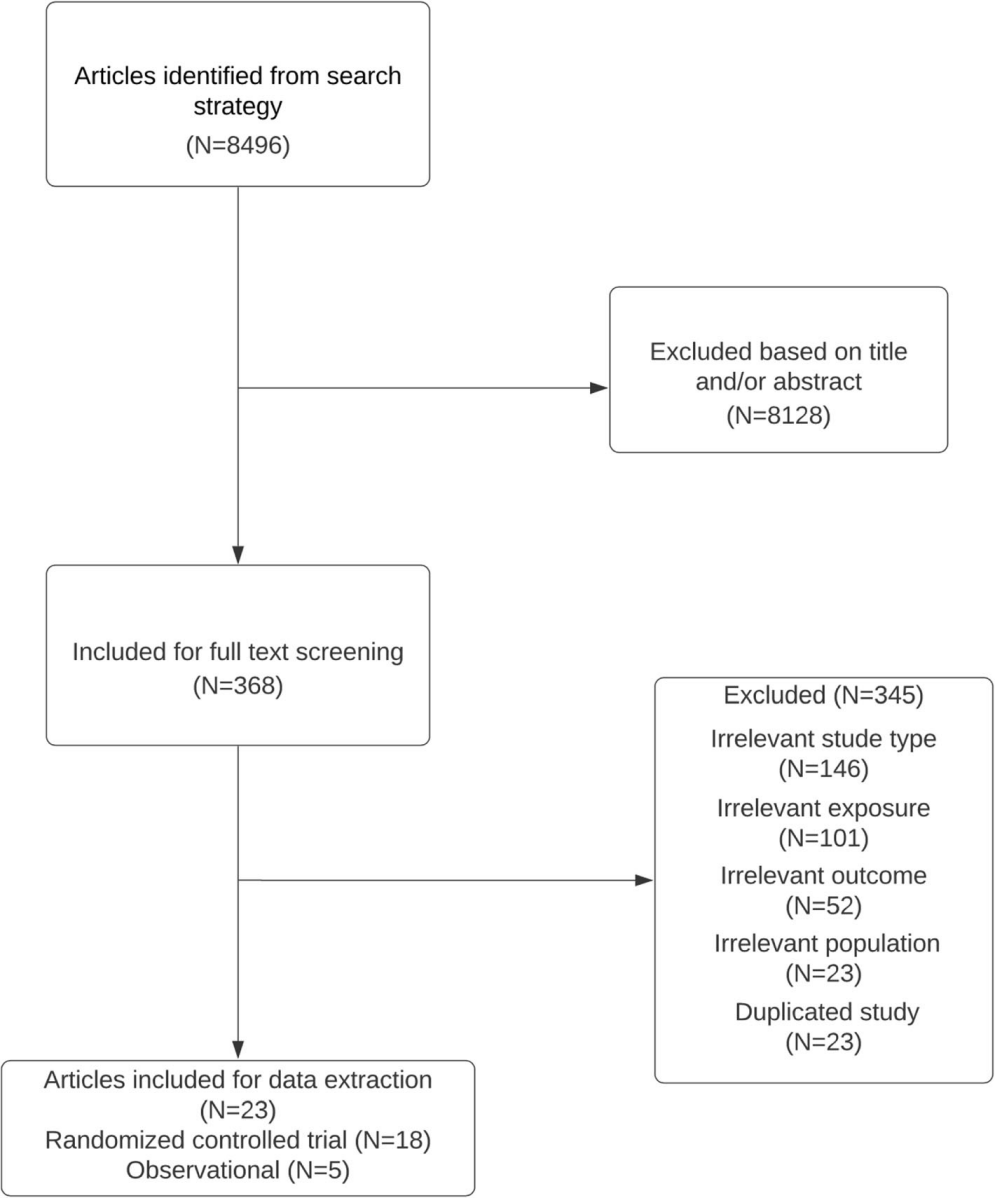
Flow chart of included studies

From 8496 retrieved references from databases, a total of 23 reports [14–16, 49–68], including eighteen RCTs [14–16, 49–61, 67, 68] and five observational studies [62–66] were included in this systematic review. Three reports [49, 52, 55] were extended results for HRQOL outcomes from main clinical trials [16, 51, 56] Therefore, fifteen independent RCTs were included. The inclusion process is depicted in Fig. 1.

### Characteristics of included RCTs

All included RCTs were parallel group trials evaluating ESA treatment for anemia reporting outcomes in physical HRQOL (Table 1) [14–16, 49–61, 67, 68]. They tested different targets, which varied from 11 to 13 g/dL to 13.5–16 g/dL for higher target arms, whereas control group targets ranged from 9.0–12.9 g/dL. Mean achieved hemoglobin levels in control groups varied from 10.2 to 11.7 g/dL, while in higher target groups the limits were 11.7 to 14 g/dL.

**Table 1:** Characteristics of included RCTs

First Author	Publication Year	Design	RRT	Control (Target)	Intervention (Target)	Pop. (n)	Mean eGFR (mL/min)	eGFR method	Instrument	% M	Age(y) mean Total (sd)	Mean baseline hemoglobin mg/dl (sd)	Follow up (weeks)	Inclusion criteria	Mean achieved Hb control (sd)		Mean achieved Hb intervention (sd)	Iron target Cointervention
Churchill, D (Canadian)	1990	Double blind RCT	Yes	9.5–11.0	11.5–13.0	78	5D	NR	SIP and KDQ	57	44(16)	7 (1.0)	24	CKD-5D stable for 3 months + Hb < 9, HD 3x week	10.2 (1.0)		11.7 (1.4)	Oral or IV iron at discretion of investigators
Parfrey, P. S.	2005	Double blind RCT	Yes	9.5–11.5	13.5–14.5	596	5D	R	KDQOL, SF-36 and FACIT	60	51 (15.4)	11 (1.2)	72	CKD incident in HD (3–18 months) + neither HF nor CHD	10.8 (1.2)		13.1 (1.5)	TSAT > 20%
Pfeffer, Ma (TREAT)	2009	Double blind RCT	No	> 9	13	4038	33	MDRD	FACIT and SF36	41	68 (60–75)^a^	10.5 (9.8–10.9)^a^	97	eGFR 20–40, diabetic, Hb < 11, TSAT > 15	10.6 (9.9–11.3)^a^		12.5 (12–12.8^a^)	NR
Roger, Sd	2014	Single blind RCT	No	> 9.5	13	51	27	MDRD	FACIT and SF36	57	80 (4.9)	10 (1.01)	24	eGFR 60 - < 15, Hb < 11, TSAT > 15	10.5 (10.1–11)^b^		12.5 (12.1–12.8)^b^	NR
Akizawa, T.	2011	Open Label	No	9–11	11–13	322	12	MDRD	FACIT and SF36	50	65 (11.8)	9.15 (0.8)	28	Hb < 10	10.0 (0.8)		11.7 (0.8)	TSAT > 20%/ferritin > 100 ng/mL
Singh, Ak (CHOIR)	2006	Open Label	No	11.3	13.5	1432	27	MDRD	KDQ and SF-36	44	66 (14.3)	10.1 (0.9)	144	Hb < 11, eGFR 15–50	11.3 (0.9)		12.5 (0.9)	NR
Drueke, T. (CREATE)	2006	Open Label	No	10.5–11.5	13–15	603	24	Cockcroft–Gault	SF-36	57	59 (14.6)	11.6 (0.6)	48	Hb < 11, eGFR 15–30	11.5 (1.0)		13 (1.0)	Oral or IV iron at discretion of investigators
Rossert, J.+	2006	Open Label	No	11.0–12.0	13–15	390	30.3	NR Cockcroft-G	SF-36 and Katz	40	58 (13.6)	11.5 (1.0)	24	CKD 25–60, TSAT > 20, ferritin > 100	12.0		14.0	NR
Villar, E	2011	Open Label	No	11.0–12.9	13.0–14.9	89	30.0	MDRD	SF-36	62	65 (8)	11.4 (0.8)	48	DM2, Hb 10–12, eGFR 25–60	11.5	13.0		Oral or IV iron at discretion of investigators (ferritin > 200mcg/l)
Furuland, H.	2003	Open Label	Both	9.0–12.0	13.5–16.0	416	NR	Iohexol-C	KDQOL	63	63 (13)	11.0 (1.0)	48	Hb 9–12, eGFR < 30 mL/min	11.5 (1.5)	13.5 (1)		TSAT > 20%,ferritin > 250 mcg/L
Besarab (Normal Hematocrit)	1998	Open Label	Yes	9.0–11.-0	13.0–15.0	1233	5D	NR	SF-36	50	65 (12)	10.1 (1.0)	72	CKD-HD,HF or IHD, TSAT > 20	10.3	13.3		TSAT> 20%
Foley, Rn	2000	Open Label	Yes	9.5–10.5	13.0–14.0	146	5D	NR	KDQOL, SF-36	62	62 (56–65)^b^	10.4	48	HD > 3 months, LV hypertrophy or dilatation, Hb 9–11	10.4 (10.2–10.6)^b^	12.2 (12.5–11.9)^b^		TSAT > 20%
Ritz, E. + (ACORD)	2007	Open Label	No	10.5–11.5	13.0–15.0	172	45	Cockcroft–Gault	SF-36	50	58 (49–69)^a^	11.9 (11.3.-12)^a^	60	CKD-DM 1 or 2, stage 1–3, Hb 10.5–13	11.5	13.0		NR
Levin+	2005	Open Label	No	9–10.5	12–14	172	28	MDRD	SF-36	70	57 (15)	11.7 (0.8)	96	CKD stages 2–4, progressive decline Hb (> 1 g/dL) within 12 m	11.7	13.0		TSAT > 20%, ferritin > 60 mcg/L
MacMahon+	2000	Double-Blind	Yes	10.0	14.0	30	NR	NR	SIP	NR	NR	8.5 (0.2)	12	Prevalent HD patients > 12 m	10.0	14.0		TSAT > 20%, ferritin > 100 mg/dL

^a^ range. ^b^ Confidence interval. + not included in the meta-analysis. *LVMI* Left Ventricular Mass Index, *LV* Left Ventricle, *CDV* Cardiovascular, *eGFR* Estimated Glomerular Filtration Rate, *CKD* Chronic Kidney Disease, *ESA* Erythropoiesis stimulating agent, *Hb* Hemoglobin, *HD* Hemodialysis, *TSAT* Transferrin Saturation, *IV* Intravenous, *NR* Not reported, *DM2* Diabetes mellitus 2, *CKD* Chronic kidney disease, *KDQOL* Kidney Disease Quality of Life Instrument. *SF-36* Short Form 36, *SIP* Sickness Impact Profile

Five studies included only hemodialysis patients [49–51, 54–56, 68], one included hemodialysis, peritoneal dialysis and non-dialysis individuals [57] and nine included only non-dialysis patients. The most frequently used questionnaire for quality of life assessment was SF-36, followed by FACIT. Inclusion criteria varied across studies, particularly regarding the decision to include only patients with specific comorbidities, e.g. heart failure and diabetes and in the limits for variables related to iron metabolism, i.e. TSAT and ferritin. Follow-up ranged from 12 to 144 weeks.

### Risk of bias assessment for RCTs

For RCTs, risk of bias differed considerably (Supplementary Figure1). Six studies had high risk of bias for incomplete data analysis criteria, mainly because patients contributing to HRQOL data were a subgroup from the randomized population, either due to attrition (e.g. high rates of loss to follow-up or mortality) or missing baseline assessments for some groups. Few studies reported assumptions for dealing with missing data, such as imputation methods, or clarified the number of patients in which HRQOL was assessed.

### Analysis of physical function and physical role

**Fig. 2:**
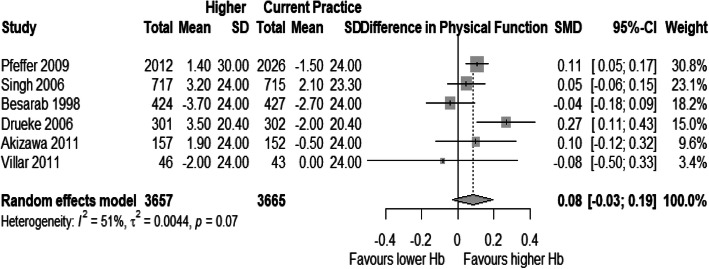
Forest plot for mean standardized difference in mean changes from baseline for Physical Function

**Fig. 3:**
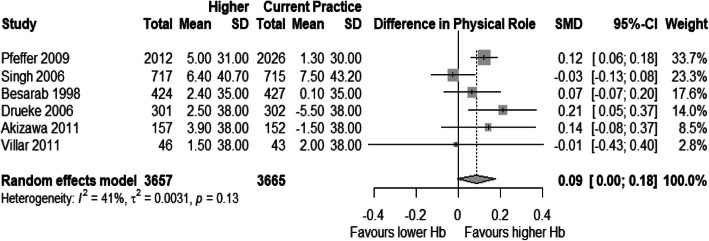
Forest plot for mean standardized difference in mean changes from baseline for Physical Role

Few trials reported data on Physical Function and Physical Role subdomains of SF-36. Thereby, only six studies contributed to analyses for both dimensions [15, 16, 52, 55, 56, 58, 60]. All studies used SF-36 for these HRQOL domains. The weighted SMD for physical function was 0.08 [95% CI: − 0.03 – 0.19] and for physical role, 0.09 [95% CI: - 0.0025 – 0.18], with a significant amount of heterogeneity in both analyses (Figs. 2 and 3).

### Analysis of fatigue domain

**Fig. 4:**
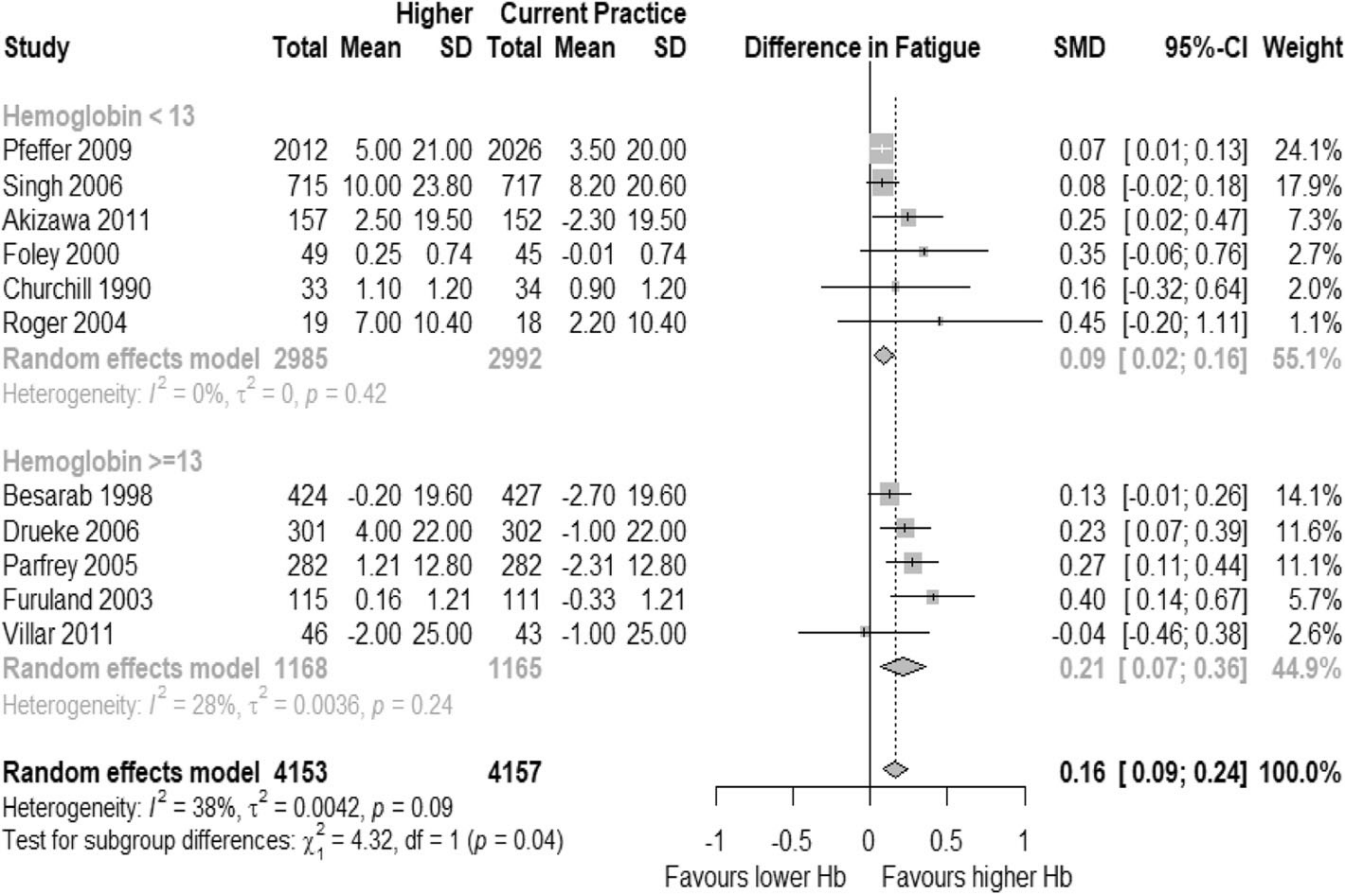
Forest plot for mean standardized difference in mean changes from baseline for Fatigue

Eleven studies were eligible for meta-analysis of fatigue [14–16, 49–58, 60]. From these, nine used SF-36 vitality subscale, and the remaining used the KDQ questionnaire. In these studies, achieved hemoglobin in control groups ranged from 10.5–11.5 g/dL, and in higher hemoglobin arms it varied from 11.7 to 13.5 g/dL. The weighted SMD between groups for fatigue was 0.16 [95% CI: 0.09–0.24], with I^2^ = 38% (Fig. 4). The point estimate, on SF-36 vitality scale, would be 3.2 [95% CI: 1.8–4.2]. Studies that reported mean achieved Hb higher or equal than 13 g/dL estimated higher effect sizes compared to lower achieved Hb studies: 0.21 [95% CI: 0.08–0.35] and 0.09 [95% CI: 0.02–0.16], respectively. The results were still significant after sensitivity analysis including only studies using SF-36 and excluding studies that required imputation.

**Fig. 5:**
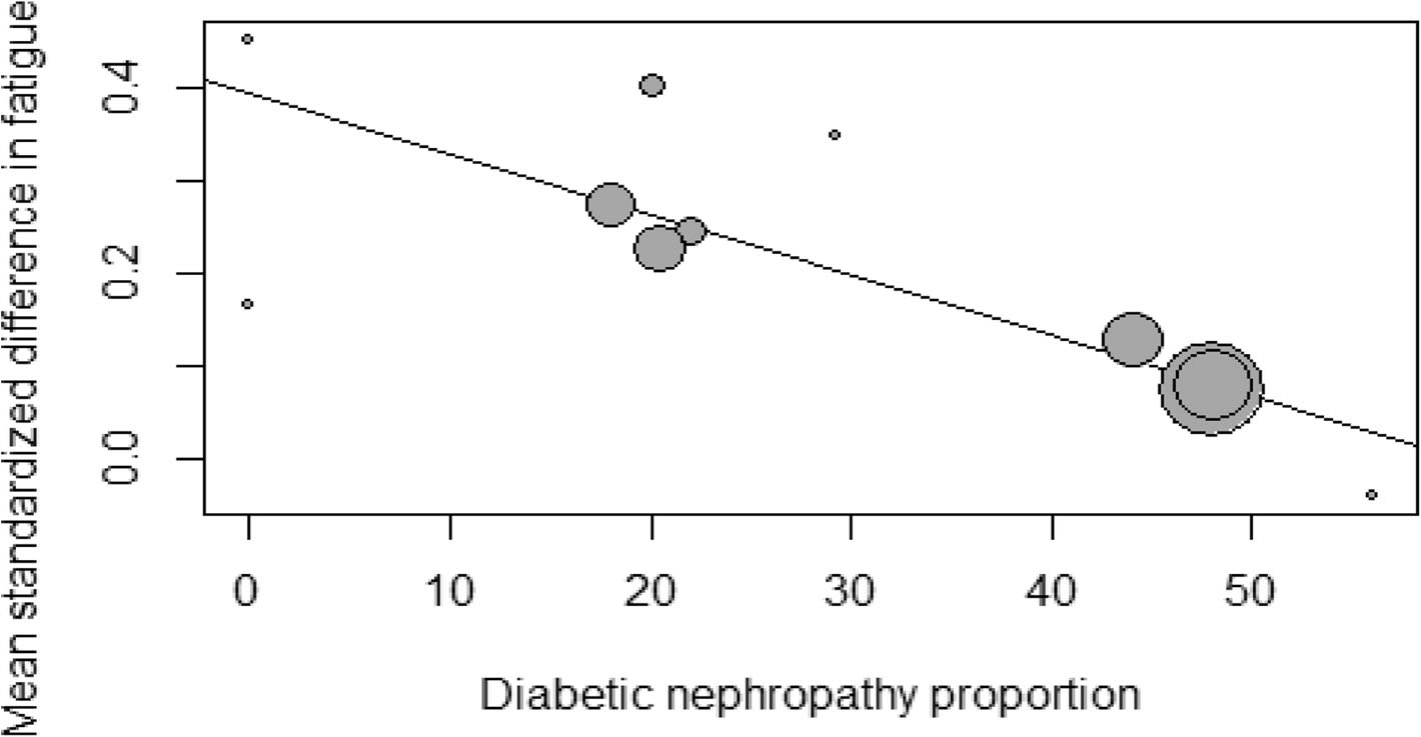
Mixed effects meta-regression of diabetic nephropathy proportion and effect sizes for mean standardized differences in fatigue

**Fig. 6:**
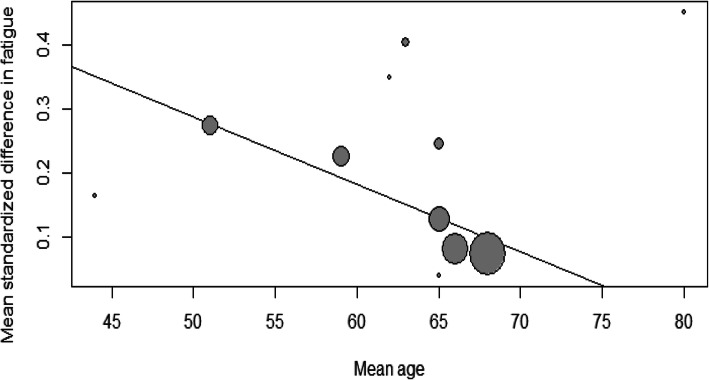
Mixed effects meta-regression of mean age and effect sizes for mean standardized differences in fatigue

Mixed effect meta-regression results are presented in Table 2. The proportion of patients with long-term complicated diabetes, mean age from included populations and follow-up time for HRQOL assessment were associated with lower estimated effect sizes in the analysis (Figs. 5 and 6). Subgroup analyses are presented in Table 3. Neither RRT nor blinding of participants and outcome evaluators were associated with effect modification.

**Table 2:** Mixed effect meta-regression for fatigue outcome

Variable	Beta	SE	Confidence Interval	*p* value
Diabetes	- 0.007	0.002	- 0.01 – - 0.003	0.0003
Follow up (weeks)	-0.002	0.0007	- 0.003 – - 0.0004	0.009
Age	-0.01	0.005	- 0.01 – -0.0007	0.03

*SE* Standard error

**Table 3:** Subgroup analysis for fatigue outcome

Variable	Yes (effect size)	Yes (n studies)	No (effect size)	No (n studies)	*P* value
Blinding	0.17	4	0.17	7	0.95
RRT	0.23	5	0.11	6	0.06

*RRT* Renal replacement therapy. Effect sizes: mean standardized differences for mean changes from baseline for Fatigue in subgroups. *P* values for Cochran’s Q test

**Fig. 7:**
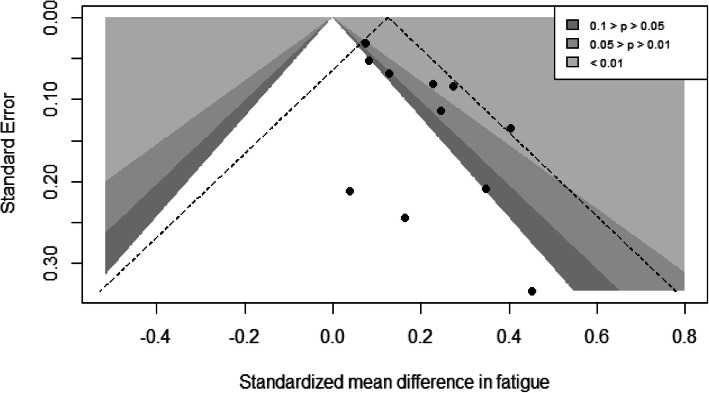
Contour funnel plot for standardized mean differences in fatigue. Shaded areas correspond to different *p* values given the standard error for assumed fixed effect

The funnel plot for the fatigue outcome (Fig. 7) suggested asymmetry on visual inspection, which was confirmed in the Egger test (*p* = 0.014). Adjusting for potential small study effects using trim and fill method, the SMD between groups was reduced to 0.10 [CI 0.01–0.19], representing 2 [95% CI: 0.2–3.8] points difference on the SF-36 vitality scale.

### Functionality

One RCT included functionality as a secondary outcome, assessed by the Katz scale [61]. Due to early termination, the study reported that analysis on ADLs was not possible given the lack of variation in this variable.

### Characteristics of observational studies

Five cohort studies were included in the systematic review (Table 4) [62–66]. Two studies evaluated the effect of different Hb levels on HRQOL outcomes [63, 65], one assessed the impact of different ESA doses and iron prescriptions on HRQOL [66], and another two approached the influence of anemia on functionality outcomes [62, 64].

**Table 4:** Characteristics of observational studies included

Study	Design	Exposure	pop. (n)	Mean eGFR (mL.min)	Age(y) mean Total	Mean Hb mg.dl (sd)	ESA use (%)	Diabetes (%)	Follow up (months)	Instrument	Population characteristics	Estimates for mean difference PCS	Estimates for mean difference fatigue	Quality score
Plantinga, L.	Prospective Cohort	Hb > 11 after 6 months starting HD	438	5D	60	10.4	70%	NR	12	SF-36	Incident HD patients, > 18 years, HRQOL collected	1.56 (0.16,2.96)	2.39 (-0.51,5.29)	8
Freburger	Retrospective Cohort	ESA dose terciles and Iron treatment	13,039	5D	59	11.8	94%	60% (42%)	12	SF-36	Prevalent HD pts., Medicare database, TSAT + HRQOL	Overall: - 0.1 (-0.7 to 0.5). Hb < 11 g/dL: 2.5 (0.6 to 4.3)	NR	6
De Goeij	Prospective Cohort	11 < Hb < 12 vs. Hb > 13	371	16.9	69 (55–76)	12.3 (1.5)	48	26 (13)	24	SF-36	Prevalent pre-dialysis patients	Overall: 4.9 * - younger/ESA 8.9 (2.1, 15.8)	5 *	8
Binder	Prospective Cohort	Anemia (WHO criteria)	311	45	84	12	0	42	26	Barthel index	Nursing residents with CKD	–	–	5
Schnelle	Retrospective Cohort	Anemia (WHO criteria)	173	40	84 (7)	NR	NR	NR	12	NR	Nursing residents with CKD	–	–	2

*HD* Hemodialysis, *TSAT* Transferrin Saturation, *WHO* World Health Organization (Hb < 12 g/dL). * Confidence interval not provided. *P* value reported as significative. *PCS* Physical Component Score, *DM* Diabetes mellitus, *Mo* Months, *CHD* Coronary heart disease. *IHD* Ischemic Heart Disease, *M* Male, *LV* Left Ventricular, *RRT* Renal replacement therapy, *NR* Not reported, *5D* Stage 5 CKD, *IV* Intravenous

Two studies included dialysis patients [65, 66], whereas the remaining one enrolled individuals not on RRT with different glomerular filtration rates. Mean age ranged from 59 to 84 years, mean hemoglobin varied from 10.4 to 12.3 g/dL and the minimum and maximum follow-up periods were 12 and 26 months respectively. The SF-36 questionnaire was the instrument used in all studies examining HRQOL. For assessments of functionality, the Barthel Index was used by one study while the ADLs were defined by the Minimum Data Set for assessment of nursing residents in the other study.

### Risk of bias in observational studies

The quality score of observational studies varied from 2 to 8 points. Two studies achieved the maximum scores on quality assessment, both investigating the effects of different achieved hemoglobin levels on HRQOL. Apart from studies addressing functionality as an outcome, all analyses were adjusted for defined sets of potential confounders, including comorbidities, sex, age, renal function and anemia treatment.

### Summary of observational studies for physical HRQOL

The two studies comparing different hemoglobin values on HRQOL outcomes were prospective studies, enrolling 809 individuals, with different patient characteristics [63, 65]. One study included incident hemodialysis individuals followed for 12 months, while the other one enrolled patients from pre-dialysis care, followed for 24 months. Both studies reported positive associations between Hb and physical HRQOL, with different effect sizes. In the study including hemodialysis patients, the mean difference in comparison groups (Hb ≥ 11 g/dL vs. Hb ≤ 11 g/dL) did not reach MID for Physical Composite Score (PCS), whereas individual subdomain differences - physical functioning and role physical - were reported to be greater than MID. Using continuous variable analyses, this study estimated a positive association between Hb and physical HRQOL with additional benefits beyond 12 g/dL. The study composed by pre-dialysis individuals reported overall effect sizes higher than MID for SF-36 in PCS and vitality, comparing patients with Hb between 11 mg/dL and 12 mg/dL with ≥13 mg/dL. The subgroup analysis in this report suggested that the benefit was greater for younger patients who received ESAs.

The retrospective study evaluating the impact of ESAs doses and iron prescription included 13,039 hemodialysis patients [66]. Overall, patients in the lowest tercile of ESA doses did not demonstrate different HRQOL for PCS score compared to individuals in the highest tercile. Subgroup analysis showed a difference between groups among patients with hemoglobin ≤11 g/dL on baseline. However, the effect size was lower than MID.

### Functionality

Two observational studies stratified patients by anemia status and estimated associations on functionality outcomes [62, 64]. None of them provided adjusted estimates for the association between anemia and limitations for ADLs. The retrospective study reported that the proportion of patients requiring assistance for ADLs was higher among anemic patients, while the prospective study estimated that the Barthel index was lower among anemic CKD individuals.

## Discussion

In this systematic review and metanalysis of real-world practices on achieved Hb, we found that fatigue - but not physical function and physical role - may be improved at hemoglobin ranges beyond the current practice recommended targets in CKD patients treated with ESAs. Our subgroup analyses and meta-regression showed that the benefit may be higher for younger patients and those free from long-term complicated diabetes. To our knowledge, this is the first evidence suggesting flexible targets for this population using only high-quality studies.

The main result of this systematic review and metanalysis was that achieved hemoglobin within 11.7–13.5 g/dL is associated with a small but clinically significant benefit for fatigue compared to current target ranges recommended by guidelines of 10–11.5 g/dL. The subgroups of RCTs reporting achieved Hb > 13 g/dL on follow-up demonstrated higher effect sizes for changes in fatigue, suggesting that incremental benefits in fatigue could be associated with higher Hb values, although the effects are modest in magnitude. This was also suggested by the prospective cohort studies included in this review, in one case particularly for younger patients treated with ESAs with Hb > 13 g/dL [63, 65].

We hypothesized that underlying factors within populations from studies would be effect modifiers [69]. Specifically, we hypothesized that conditions leading to disability, e.g. age and comorbidities, would reduce the impact of hemoglobin on physical HRQOL [63, 70] . Our analysis seems to support this model, since both age and proportion of patients with long-term diabetes - important predictors of disability [71] - were associated with lower effect sizes for Hb differences on fatigue.

Importantly, different assumptions about the relations between variables could lead to distinct interpretations. For instance, achieved Hb could be seen as a mediator of the association between age/comorbidities and improvements in fatigue at higher than current target Hb levels, for these variables are predictors of ESA responsiveness [72, 73]. Another possibility is that age and proportion of patients with long-term diabetes are proxies for underlying risks within these populations when exposed to ESAs, which would lead to negative effects on fatigue through cardiovascular outcomes [73, 74]. Additionally, longer follow-up time for HRQOL assessment was associated with lower effect sizes. This might suggest that the effect of Hb on fatigue may reduce over time, which could result from adverse effects from ESAs or the progressive disability over time [51].

The subset of studies evaluating functionality had generally low quality and demonstrated that the association between disability on ADLs and hemoglobin remains underexplored in well-designed studies [62, 64]. Independency is one of the most important outcomes for patients and families [1, 75], and CKD has been associated with worse functional outcomes, particularly after starting RRT [76].

Previous systematic reviews evaluated the impact of ESAs on general HRQOL, including physical components [20–22]. Collister and colleagues estimated that the effect on several HRQOL domains of patients treated for higher targets with ESAs are lower than MID for SF-36 compared to lower targets [21]. In that study, they included reports that compared both different ESAs targets and ESA versus placebo with no rescue strategy, therefore including a subgroup of patients with severe anemia [21]. Achieved Hb ranges for higher and lower targets groups overlapped and the resulting estimates were highly heterogeneous, as acknowledged by the authors [21]. As achieved Hb differs from designed target for a set of RCTs, probably because of different distributions of ESA responsiveness and adherence to protocol, clinical interpretation of aggregate estimates in this context may be difficult. By design, our study provided estimates contrasting different achieved Hb to provide clear counterfactuals.

The current boundaries for hemoglobin values are defined for safety reasons that were specifically based on the ESA trials [11]. However, along with the development of new drugs for treatment of CKD anemia, such as the recently published trials on the HIF-stabilizers [77, 78], and the advances in characterizing distinct populations at risk, a renewed approach toward flexible targets could lead to modest, but important benefits for patients on fatigue outcomes [79, 80].

This study presents important limitations. Clinically, the Hb ranges we set to study are not recommended in current clinical practice, given the well-established risks associated with aiming Hb targets beyond 11.5–12 g/dL with available interventions [11]. In light of the person-centered value care paradigm, some patients may accept higher risks aiming to attain better physical HRQOL [8], thereby yielding greater autonomy in an informed-decision model [2, 3]. However, our study did not provide additional information on risks associated higher Hb targets, which have been summarized in other studies [13]. Moreover, ESAs effects on physical HRQOL could occur through different significant mediators, which would limit the inferences from the estimates we presented. For instance, the magnitude of the impact of cardiovascular events caused by ESAs on HRQOL, particularly for fatigue, and the extension to which they are independent of Hb variation remains unknown [74, 81]. However, under the hypothesis that non-erythropoietic effects of ESAs could be associated with lower physical HRQOL mediated by cardiovascular events [82, 83], the pooled estimates for Hb effects would presumably be underestimated.

Some points about fatigue assessment should be acknowledged, which is the subdomain of physical HRQOL more associated with anemia and CKD [36]. Fatigue is a multidimensional concept that is assessed by distinct instruments and each one may evaluate a diverse set of dimensions [5, 36]. We only evaluated, for the meta-analysis, studies using KDQOL/SF-36 and KDQ questionnaires, which have been both shown to measure the same dimensions within the fatigue framework but may have different psychometric properties [36]. Remarkably, the SF-36 vitality scale, although often used both in RCTs and observational studies, has been shown to lack content validity for hemodialysis patients [36]. This raises questions about the meaning of fatigue assessment in this population [36]. The development of broader, more reliable and valid instruments for CKD patients and especially for anemia symptoms may provide better consistency in estimates for clinical practice [84].

Methodologically, some limitations should be further noted. We could not define subsets of studies according to strict Hb ranges. However, the achieved Hb levels in control groups in ESA target trials reflects current clinical practice recommendations for anemia management [11]. Studies often assessed physical HRQOL in subgroups of the randomized population [85]. Imputation assumptions were often not declared and any association between the frail population within these studies, with higher rates of missing data, and effect size would lead to distinct estimates from the true effect [85]. Under the assumption that frail patients benefit less from hemoglobin differences on physical HRQOL the estimates presented here would be oversized. Additionally, we extracted the last reported valued on follow-up when multiple assessments of physical HRQOL were available, as the interest of the analysis was on sustained effects on real-world achieved Hbs resulting from ESA intervention. This decision, although consistent with a more pragmatic approach for measuring the effects we were interested in, could result in more heterogeneity in the effect estimates. Finally, our meta-analysis was restricted to the population of CKD patients receiving ESAs, therefore our results could not be generalized beyond this subgroup.

Our study shows relevant strengths. We included only studies in which populations are representative of current clinical practice in terms of Hb ranges, in order to provide meaningful counterfactuals for Hb differences in physical HRQOL estimates, thereby assessing potential efficacy of achievement of Hb on these outcomes. As a consequence, our estimates presented low to moderate heterogeneity, particularly for fatigue. We could further explore the heterogeneity in distinct subgroups according to pre-specified variables dictated by an a priori defined model. We also provided estimates for publication bias for fatigue outcomes, which demonstrated significant small study effects in this literature.

## Conclusions

In summary, achieved hemoglobin higher than currently recommended targets may be associated with relatively modest improvement in fatigue. The lack of robustness to publication bias adds a limitation to the significance of our findings, which should be interpreted with caution. Younger and non-diabetic patients may experience more pronounced benefits of higher Hb levels after treatment with ESAs [11]. The present study suggests the recommendation for individualization of anemia management may yield improvements on the patient prioritized fatigue symptom [11]. Further intervention studies evaluating risks and benefits of higher achieved Hb on physical HRQOL for subgroup of patients with lower risk of adverse events, particularly with new interventions for CKD anemia, are needed to provide better information for patient-centered informed decisions on clinical management of CKD anemia.

## Supplementary information

**Additional file 1.**

## Data Availability

The datasets used and/or analysed during the current study are available from the corresponding author on reasonable request.

## Abbreviations

LVMI: Left Ventricular Mass Index
LV: Left Ventricle
CDV: Cardiovascular
eGFR: Estimated Glomerular Filtration Rate
CKD: Chronic Kidney Disease
ESA: Erythropoiesis stimulating agent
Hb: Hemoglobin
HD: Hemodialysis
TSAT: Transferrin Saturation
IV: Intravenous
NR: Not reported
DM2: Diabetes mellitus 2
CKD: chronic kidney disease
KDQOL: Kidney Disease Quality of Life Instrument
SF-36: Short Form 36
SIP: Sickness Impact Profile
