# Supplementary material for: Physical health-related quality of life at higher achieved hemoglobin levels among chronic kidney disease patients: a systematic review and meta-analysis
Source: BMC Nephrol. 2020 Jul 8;21:259. doi: 10.1186/s12882-020-01912-8 (PMC7346455; doi:10.1186/s12882-020-01912-8)
Supplement: Supplementary file 1 — Additional file 1. [file 12882_2020_1912_MOESM1_ESM.docx]

**Supplementary Material**

| **First Author** | | **Design** | **Primary Outcome** | **EPO used** | Baseline physical HRQOL | | | | | | | | | | | | |  | |  | |  |  |  |  | |  | | |  |  | |  | |  | |  | |  |  |  |  |  |
| --- | --- | --- | --- | --- | --- | --- | --- | --- | --- | --- | --- | --- | --- | --- | --- | --- | --- | --- | --- | --- | --- | --- | --- | --- | --- | --- | --- | --- | --- | --- | --- | --- | --- | --- | --- | --- | --- | --- | --- | --- | --- | --- | --- |
|  |  |  |  |  | **FACT cont.** | **FACT int.** | **SF-36 vitality cont.** | **SF-36 vitality int.** | **SF-36 Physical Role cont.** | **SF-36 Physical Role int.** | **SF-36 Physical Functioning cont.** | **SF-36 Physical Functioning int.** | **physical KDQOL** | **physical KDQOL** | **Fatigue HRQOL (mean) cont.** | **Fatigue HRQOL (mean) int.** | **Iron use (%)** | | **Ferritin** | | **TST** | **Diabetes (%)** | | **HF (%)** | | **Catheter (%)** | | **Time on dialysis (yr)** | **Kt/V** | | | **Follow up** | | **Prop IV iron prescript. cont. (%)** | | **Prop IV iron prescript. int. (%)** | | **Main Conclusions** | | | | | |
|  |  |  |  |  |  |  |  |  |  |  |  |  | **(mean) Cont.** | **(mean) Int.** |  |  |  |  | **(sd)** | | **(%)** |  |  |  |  |  |  |  |  |  |  | **(months)** | |  |  |  |  |  |  |  |  |  |  |
| Churchill, D | | double blind RCT | Six-Minute Walk Test | EPO | - | - | - | - | - | - | - | - | 3.6 | 3.9 | 4.1 | 4.2 | NR | | 1100(1600) | | NR | 0 | | NR | | - | | - | - | | | 6 | | 35 | | 58 | | EPO improves QOL vs. Placebo, but not across EPO targets | | | | | |
| Parfrey, P. S. | | double blind RCT | Left Ventricular Vol. Index | EPO | NR | NR | NR | NR | - | - | - | - | - | NR | NR | NR | NR | | NR | | 36.2 | 18 | | 0 | | - | | - | - | | | 24 | | 25 | | 37 | | Higher Hb target doesn't change LV index or incidence of HF | | | | | |
| Pfeffer, Ma | | double blind RCT | general and CDV mortality | DP | 30.2 | 30.4 | 44.9 (22) | 46.9 ( 23) | 47.5 ( 28) | 47.9 (30) | 44.9 (26) | 45 (27) | - | - | - | - | 42 | | 134 | | 23 | 100 | | 33 | | - | | - | - | | | 38 | | 20.4 | | 14.8 | | Higher Hb target raised cerebrovascular and thrombotic risk | | | | | |
| Roger, Sd | | single blind RCT | Vitality on SF-36 | DP | - | - | 44.5 | 44.4 | - | - | - | - | - | - | - | - | NR | | 235(220) | | 24(8) | 0 | | NR | | - | | - | - | | | 9 | | NR | | NR | | ESA do not improve vitality among elderly CKD patients | | | | | |
| Akizawa, T. | | Open Label | SF-36 and LVMI | EPO-DP | - | - | 65 (20.4) | 63.4 (21.7) | 80.9 (22.8) | 77.2 (26.7) | 76.1 (19) | 73.3 (22.4) | - | - | - | - | NR | | 130 (191) | | 30 (12) | NR | | 7.5 | | - | | - | - | | | 12 | | NR | | NR | | Higher Hb associated with better outcomes on LVMI and HRQOL | | | | | |
| Singh, Ak | | Open Label | Composite outcome CDV | EPO | - | - | 36.6 ( 22.4) | 35.2 (22.6) | 32.5 (29.2) | 31.9 (38.9) | 42.4 (27.3) | 41.9 (28.2) | - | - | - | - | 26.5 | | 173 (164) | | 25 (10.5) | NR | | 24 | | - | | - | - | | | 36 | | NR | | NR | | Higher risk for CDV events in higher Hg group. More important in AHF events | | | | | |
| Drueke, T. B | | Open Label | CDV events | EPO | - | - | - | - | - | - | - | - | - | - | - | - | NR | | 181.9 (153) | | 31.8 | 27 | | 33 | | - | | - | - | | | 24 | | NR | | NR | | EPO doesn't reduce CVD events. May improve QOL. | | | | | |
| Rossert, J. | | Open Label | Progression CKD | EPO | - | - | - | - | - | - | - | - | - | - | - | - | NR | | NR | | NR | 34 | | NR | | - | | - | - | | | 7 | | NR | | NR | | EPO may improve QOL. | | | | | |
| Ritz, E. | | Open Label | LVMI | EPO | - | - | - | - | - | - | - | - | - | - | - | - | NR | | 112 | | 22 | 69 | | 3.5 | | - | | - | - | | | 15 | | NR | | NR | | EPO doesn't change LVMI, may improve QOLF | | | | | |
| Villar, E | | Open Label | Progression CKD | EPO-DP | - | - | 39 | 41 | 47 | 46 | 50 | 50 | - | - | - | - | NR | | 167.5 (182) | | NR | 100 | | NR | | - | | - | - | | | 24 | | 0 | | 0 | | EPO doesn't reduce CKD progression. No impact in QOL either. | | | | | |

|  |  |  |  |  |  |  |  |  |  |  |  |  |  |  |  |  |  |  |  |  |  |  |  |  |  |  |  |
| --- | --- | --- | --- | --- | --- | --- | --- | --- | --- | --- | --- | --- | --- | --- | --- | --- | --- | --- | --- | --- | --- | --- | --- | --- | --- | --- | --- |
|  |  |  |  |  |  |  |  |  |  |  |  |  |  |  |  |  |  |  |  |  |  |  |  |  |  |  |  |
| Besarab , Anatole | Open Label | AMI or death | EPO | - | - | 44.7 ( 21.8) | 44.5 ( 22) | 33.3 (38.5) | 33.1 (38.9( | 38.6 (27.5) | 38.6 (26.7) | - | - | - | - | NR | NR | NR | 56 | 100 | 10 | 3.1 | 1.38 | 36 | 75 | 85 | Higher Hb may increase mortality, vascular thrombosis, and QOL |
| Foley, Rn | Open Label | LVVI | EPO | - | - | - | - | - | - | - | - | 3.68 (3.28,4.08) | 3.49 (3.17,3.81) | 4.53 (4.15,4.91) | 4.41 (4.10,4.73) | NR | NR | NR | NR | 100 | 7 | 4.6 | 1.48 | 12 | NR | NR | Higher Hb doesn't improve echocardiographic parameter. May improve QOL |
| Furuland,H | Open Label | HRQOL | EPO | - | - | - | - | - | - | - | - | 4.0(1-7) | 3.83 (1.6-7) | 5 (1-7) | 4.6 (1.17-7) | NR | NR | NR | 20 | 15.5 | - | - | - | 12 | 33 | 59.3 | EPO improves HRQOL. Reaching specified target may reduce mortality |
| Levin | Open Label | LVMI | EPO | - | - | - | - | - | - | - | - | - | - | - | - | 80 | 100 (60-172) | 27(10) | 38 | - | - | - | - | 24 | 11 | 13 | LVMI may not be affected by early anemia treatment |
| McMahon | Double-Blind | NR | EPO | - | - | - | - | - | - | - | - | - | - | - | - | - | - | - | - | - | - | - | - | - | - | - | Higher Hb may improve LVMI and HRQOL |

| **First Author** | | **Design** | **Primary Outcome** | **ESA used** | Baseline physical HRQOL | | | | | | | | | | | | |  | |  | |  |  |  |  | |  | |  |  | |  | |  | |  | |  |  |  |  |  |
| --- | --- | --- | --- | --- | --- | --- | --- | --- | --- | --- | --- | --- | --- | --- | --- | --- | --- | --- | --- | --- | --- | --- | --- | --- | --- | --- | --- | --- | --- | --- | --- | --- | --- | --- | --- | --- | --- | --- | --- | --- | --- | --- |
|  |  |  |  |  | **FACT cont.** | **FACT int.** | **SF-36 vitality cont.** | **SF-36 vitality int.** | **SF-36 Physical Role cont.** | **SF-36 Physical Role int.** | **SF-36 Physical Functioning cont.** | **SF-36 Physical Functioning int.** | **physical KDQOL** | **physical KDQOL** | **Fatigue HRQOL (mean) cont.** | **Fatigue HRQOL (mean) int.** | **Iron use (%)** | | **Ferritin** | | **TST** | **Diabetes (%)** | | **HF (%)** | | **Catheter ( %)** | | **Time on dialysis (yr)** | **Kt/V** | | **Follow up** | | **Prop IV iron prescript. cont. (%)** | | **Prop IV iron prescript. int. (%)** | | **Main Conclusions** | | | | | |
|  |  |  |  |  |  |  |  |  |  |  |  |  | **(mean) Cont.** | **(mean) Int.** |  |  |  |  | **(sd)** | | **(%)** |  |  |  |  |  |  |  |  |  | **(months)** | |  |  |  |  |  |  |  |  |  |  |

Supplementary table 1 : characteristics of included RCTs. EPO : Erythropoeitin. HF : Heart Failure. LV : Left Ventricular. CDV : cardiovascular. LVMI : Left Ventricular Mass Index. QOL : quality of life.

Search Strategy: MEDLINE

(((chronic kidney disease[mesh] OR chronic kidney disease [tiab] OR nephropathy [tiab] OR chronic renal insufficiency[mesh] OR diabetic nephropathies[mesh] OR hypertensive nephropathy[mesh] OR diabetic kidney*[tiab] OR kidney dis*[tiab] OR chronic renal failure[mesh])) AND (anemia[mesh] OR anemia[tiab] OR anemia [tw] OR hemoglobin[mesh] OR erythropoietin[mesh] OR epoetin alfa [mesh] OR epoetin beta [tw] OR epoetin*[tw] OR darbepoetin alfa [mesh] OR blood transfusion [mesh] OR hematocrit [tw] OR blood cells [tw])) AND ((functionality [tiab] OR physical function [tiab] OR fatigue [mesh] OR fatigue [tiab] OR quality of life [tiab] OR daily living activities [mesh] OR Lawton[tw] OR Katz[tw] OR chronic limitation of activity [mesh] OR independent living [mesh] OR disability evaluation [mesh] OR exercise [mesh] OR physical exertion [mesh] OR quality of life [mesh] OR well-being [tw] OR functionality[tw] OR independence [tw] OR dysfunction[tw] OR disability[tw] OR International Classification of Functioning, Disability and Health[mesh] OR SF-36[tw] OR KDQ[tw] OR accelerometer[tw] OR physical health[tiab] OR movement[tiab] OR range of motion[tiab] OR daily steps[tiab] OR steps[tw] OR mobility[tw] ))

Search Strategy: EMBASE

('chronic kidney failure'/exp OR 'chronic kidney disease' OR 'chronic kidney disorder' OR 'chronic kidney failure' OR 'chronic kidney insufficiency' OR 'chronic nephropathy' OR 'chronic renal disease' OR 'chronic renal failure' OR 'chronic renal insufficiency' OR 'kidney chronic failure' OR 'kidney disease, chronic' OR 'kidney failure, chronic' OR 'kidney function, chronic disease' OR 'renal insufficiency, chronic' OR 'kidney disease'/exp OR 'disease, kidney' OR 'kidney disease' OR 'kidney diseases' OR 'kidney disorder' OR 'kidney pathology' OR 'nephropathy' OR 'perinephritis' OR 'perirenal infection' OR 'renal disease' OR 'renal disorder' OR 'unilateral kidney disease' OR 'diabetic nephropathy'/exp OR 'kimmelstiehl wilson syndrome' OR 'diabetes nephropathy' OR 'diabetic glomerulopathy' OR 'diabetic glomerulosclerosis' OR 'diabetic intercapillary glomerulosclerosis' OR 'diabetic kidney disease' OR 'diabetic nephropathies' OR 'diabetic nephropathy' OR 'diabetic nephrosclerosis' OR 'glomerulonecrosis, intercapillary' OR 'glomerulosclerosis, diabetic' OR 'glomerulosclerosis, intercapillary' OR 'intercapillary glomerulosclerosis' OR 'kimmelstiel wilson disease' OR 'kimmelstiel wilson nephropathy' OR 'kimmelstiel wilson syndrome' OR 'nephropathy, diabetic' OR 'hypertensive nephropathy'/exp) AND ('anemia'/exp OR 'anaemia' OR 'anaemia gravis' OR 'anemia' OR 'anemia gravis' OR 'chronic anaemia' OR 'chronic anemia' OR 'infant anaemia' OR 'infant anemia' OR 'normoblastic anaemia' OR 'normoblastic anemia' OR 'secondary anaemia' OR 'secondary anemia' OR 'simple anaemia' OR 'simple anemia' OR 'erythropoietin'/exp OR 'erthropoietin' OR 'erythropoiesis stimulating factor' OR 'erythropoietic factor' OR 'erythropoietic stimulation factor' OR 'erythropoietin' OR 'hematopoietin' OR 'hemopoietin' OR 'kidney erythropoietic factor' OR 'renal erythropoietic factor' OR 'recombinant erythropoietin'/exp OR 'novel erythropoiesis stimulating protein'/exp OR 'nesp' OR 'aranesp' OR 'aranest' OR 'darbepoetin' OR 'darbepoetin alfa' OR 'darbepoetin alpha' OR 'darbepoietin' OR 'darbepoietin alfa' OR 'darbepoietin alpha' OR 'darbopoetin' OR 'darbopoetin alfa' OR 'darbopoetin alpha' OR 'darbopoietin' OR 'darbopoietin alfa' OR 'darbopoietin alpha' OR 'krn 321' OR 'krn321' OR 'nespo' OR 'novel erythropoiesis stimulating protein' OR 'blood transfusion'/exp OR 'iron deficiency anemia'/exp OR 'anaemia, hypochromic' OR 'anaemia, iron deficiency' OR 'anaemia, iron-deficiency' OR 'anaemia, microcytic hypochromic' OR 'anemia, hypochromic' OR 'anemia, iron deficiency' OR 'anemia, iron-deficiency' OR 'anemia, microcytic hypochromic' OR 'ferriprive anaemia' OR 'ferriprive anemia' OR 'hypochrome anaemia' OR 'hypochrome anemia' OR 'hypochromic anaemia' OR 'hypochromic anemia' OR 'hypochromic iron deficiency anaemia' OR 'hypochromic iron deficiency anemia' OR 'hypochromic microcytic anaemia' OR 'hypochromic microcytic anemia' OR 'hypoferrous anaemia' OR 'hypoferrous anemia' OR 'iron deficiency anaemia' OR 'iron deficiency anemia' OR 'iron deficient anaemia' OR 'iron deficient anemia' OR 'iron refractory anaemia' OR 'iron refractory anemia' OR 'microcytic hypochromic anaemia' OR 'microcytic hypochromic anemia' OR 'sideropenic anaemia' OR 'sideropenic anemia') AND ('accelerometer'/exp OR '3dnx' OR 'adxl322' OR 'actical' OR 'dynaport minimod' OR 'egas (device)' OR 'genea' OR 'gt1m' OR 'gt3x' OR 'minimod' OR 'rt3 (device)' OR 'accelerometer' OR 'accelerometers' OR 'meter, accelero' OR 'adl disability'/exp OR 'quality of life'/exp OR 'daily life activity'/exp OR 'adl (activities of daily living)' OR 'activities of daily living' OR 'activity, daily living' OR 'daily life activity' OR 'daily living activity' OR 'lawton instrumental activities of daily living scale'/exp OR 'lawton iadl' OR 'lawton instrumental activities of daily living' OR 'lawton instrumental activities of daily living scale' OR 'lawton-brody scale' OR 'katz index'/exp OR 'katz adl index' OR 'katz index' OR 'katz index of adl' OR 'katz index of activities of daily living' OR 'independent living'/exp OR 'exercise'/exp OR 'biometric exercise' OR 'effort' OR 'exercise' OR 'exercise capacity' OR 'exercise performance' OR 'exercise training' OR 'exertion' OR 'fitness training' OR 'physical conditioning, human' OR 'physical effort' OR 'physical exercise' OR 'physical exertion' OR 'restraint, physical' OR 'functionality or' OR 'functional status assessment'/exp OR 'functional status assessment' OR 'international classification of functioning, disability and health'/exp OR 'short form 36'/exp OR 'physical performance'/exp OR 'sickness impact profile'/exp OR 'sickness impact profile')

Quality Score for observational studies: Modified NewCasttle Ottawa scale.

1. Study design

0 Longitudinal retrospective study (data records)

1 Longitudinal prospective study

1. Representativeness

0 points if included patients from a single center

1 point if included patients from multicenter cohort

1. Ascertainment of Exposure

0 if exposition period not specified

1 if exposition period specified

2) Comparability :

0 points if not adjusted

1 point if adjusted for sex, age and renal function or CKD category

2 points if adjusted for sex, at least one comorbidity , age and renal function

3 points if adjusted for sex, at least one comorbidity, age, renal function and anemia treatment

3) Outcome

0 points if outcome assessments method was not described

1 point if outcome assessments were retrieved from medical records abstraction

2 points if outcome assessments were evaluated by study protocol

|  |  | Random sequence generation | Allocation concealment | Blinding of participants | Incomplete outcome data | Selective reporting |
| --- | --- | --- | --- | --- | --- | --- |
|  | |  |  |  |  |  |
|  | |  |  |  |  |  |
|  | |  |  |  |  |  |
|  | |  |  |  |  |  |
|  | |  |  |  |  |  |
|  | |  |  |  |  |  |
|  | |  |  |  |  |  |
|  | |  |  |  |  |  |
|  | |  |  |  |  |  |
|  | |  |  |  |  |  |
|  | |  |  |  |  |  |
|  | |  |  |  |  |  |
|  | |  |  |  |  |  |
|  | |  |  |  |  |  |
|  | |  |  |  |  |  |

Supplementary Figure 1 : quality of RCTs according to Cochrane tool for risk of bias in RCTs.
